# Perceptions of treatment for tics among young people with Tourette syndrome and their parents: a mixed methods study

**DOI:** 10.1186/s12888-015-0430-0

**Published:** 2015-03-11

**Authors:** José Cuenca, Cris Glazebrook, Tim Kendall, Tammy Hedderly, Isobel Heyman, Georgina Jackson, Tara Murphy, Hugh Rickards, Mary Robertson, Jeremy Stern, Penny Trayner, Chris Hollis

**Affiliations:** Division of Psychiatry and Applied Psychology, School of Medicine, Institute of Mental Health, University of Nottingham, Jubilee Campus, Nottingham, UK; National Collaborating Centre for Mental Health, Royal College of Psychiatrists, London, UK; Department of Paediatric Neurology, King’s College Hospital NHS Foundation Trust, London, UK; Department of Child and Adolescent Mental Health, Great Ormond Street Hospital for Children, London, UK; Department of Clinical Neuropsychology, Great Ormond Street Hospital for Children NHS Foundation Trust, London, UK; Department of Neuropsychiatry, University of Birmingham and Barberry National Centre for Mental Health, Birmingham, UK; Department of Neurology, St George’s Hospital and Medical School, London, UK; Division of Clinical Psychology, University of Manchester, Manchester, UK

**Keywords:** Tourette syndrome, Tics, Treatment, Young people, Parents, Qualitative analysis

## Abstract

**Background:**

Tourette syndrome (TS) among young people is associated with psychosocial difficulties and parents play an important role in the management of the condition. Clinical guidelines have been developed for the treatment of TS and tics, but little is known about how young people and their parents perceive their treatment options or their desired outcomes of treatment. The aim of this study is to explore perceptions of treatments for tics among young people with TS and their parents.

**Methods:**

In-depth interviews with 42 young people with TS and a mixed-methods, online survey of 295 parents of young people with TS. Participant recruitment was conducted through Tourettes Action (TA): a non-profit UK organisation for the support of people with TS. Interview transcripts were analysed using thematic analysis and responses to survey open-ended questions were analysed using content analysis. Triangulation of qualitative and quantitative data from the parents’ survey and qualitative data from the interviews with young people was used to increase the validity and depth of the findings.

**Results:**

A strong theme was the perception that health professionals have limited knowledge of TS and its treatment. Medication was a common treatment for tics and both young people and parents described benefits of medication. However, adverse effects were frequently described and these were a common reason for stopping medication among young people. Aripiprazole was viewed most positively. Access to behavioural interventions for tics was limited and 76% of parents wanted this treatment to be available for their child. Some young people had reservations about the effectiveness or practicality of behavioural interventions. Reduction and abolition of tics were desired outcomes of treatment, but both parents and young people also identified the importance of increasing control over tics and reducing anxiety-related symptoms. For young people, managing the urge to tic was an important outcome of treatment.

**Conclusions:**

The results suggest a need for more training in the identification and management of TS and wider availability of behavioural treatments. Clinical trials could explore the effectiveness of Aripiprazole used in combination with psycho-educational interventions to reduce anxiety and promote a sense of control.

**Electronic supplementary material:**

The online version of this article (doi:10.1186/s12888-015-0430-0) contains supplementary material, which is available to authorized users.

## Background

Previously thought of as a rare condition, the prevalence of Tourette syndrome (TS) in young people aged five to 18 years is now estimated to be between 0.4% and 3.8% with an overall prevalence of 1% [1]. TS is defined by multiple motor tics and the presence of at least one vocal tic over a period of one year [2]. Some young people with TS have been found to experience emotional difficulties, to feel different or abnormal because of tics [3] and to report problems relating with peers [4]. They are also likely to face educational problems and [5] parents of young people with TS report difficulties with their child’s behaviour [6], with both of these difficulties exacerbated by co-occurring conditions such as attention deficit hyperactivity disorder [7,5].

Some young people with TS require treatment to help them manage their tics and the associated psychosocial impairment [8]. Medication is the most common form of treatment for tics [9,10], particularly risperidone, clonidine and aripiprazole, among children and adolescents [11]. Behavioural interventions for tics such as habit reversal are used more rarely [10] although current clinical guidelines recommend that behavioural interventions for tics are given as a first-line treatment [12-14] and medication when tics are severe and distressing [13]. Current guidelines also emphasise the importance of providing information about the condition to patients and family [13,15,16]. Despite data on treatment utilisation patterns and recent clinical recommendations, little is known about how young people and key carers feel and think about different treatments for tics.

The few studies that have explored young people’s and parents’ perceptions of treatments for tics have mainly focussed on the diagnostic process or on barriers to individual treatments. A previous study of young people with TS found a median delay of 2.8 years from age of tic onset to age of diagnosis of TS [17]. Young people with TS and their parents recently interviewed in a Spanish study described difficulties receiving a diagnosis of TS that were associated with a lack of knowledge regarding tics among health professionals [18]. Furthermore, a survey study found that a common barrier for treatment of tics was difficultiy finding knowledgeable treatment providers [10]. There is some evidence suggesting that parents of young people with TS have concerns about adverse effects of medication for tics such as antipsychotics [19].

The aim of this study was to explore how young people with TS and parents of young people with TS perceive different treatment strategies for tics, and to explore similarities and differences between the views of parents and young people. We integrated qualitative data from in-depth interviews with young people with TS, with quantitative and qualitative data from an online survey of parents of young people with TS. Combining this evidence together increased the depth of the findings and permitted a better understanding of how young people with TS and their parents feel and think about their treatment options. This research was part of a larger project examining the benefits and risks of different types of treatment for tics in young people with TS (Health Technology Assessment reference 10/142/01).

## Methods

### Participants and recruitment

The study had research ethics committee approval from the Medical School Research Ethics Committee of the University of Nottingham (reference number H07062012).

#### Survey of parents

Survey participants were parents or carers of young people with TS or any tic disorder (referred to as “parents” in the remainder of this paper). Exclusion criterion was being a parent or carer of a young person aged over 17 years. Participants were recruited through study adverts that were posted on the UK Tourettes Action (TA) web site [20] and on its electronic newsletter. In addition, invitation emails that included a link to the survey were sent by TA to its members, and study leaflets were distributed at TA events such as conferences and group meetings. Participants gave online consent to participate, the survey was anonymous and it was available for six consecutive months (from December 2012 to May 2013).

#### Interviews with young people

Participants were children and young people with TS between 10 and 17 years of age (referred to as “young people” in the remainder of this paper). Recruitment was conducted through the online survey of parents, study announcements posted in TA’s website and social media, and through study leaflets distributed at TA’s informational events. Young people whose parents did not participate in the survey were still eligible to take part in the interviews.

Young people between 16 and 17 years of age, and parents of young people aged 10 to 15 years, were contacted to discuss the study in more detail, sent the information sheet in print, a consent form and a freepost envelope. Interviews were carried out at least one week after the initial contact. On the day of the interview, parents of young people aged 10 to 15 years were asked to provide verbal informed consent, which was digitally recorded with their permission. In addition, after answering any questions about the study, their children were asked to provide verbal consent to take part in the study and this was digitally recorded. Young people between 16 and 17 years of age provided verbal informed consent on the day of the interview.

The target sample size for the interviews was flexible and aimed to include up to 50 young people depending on the number of participants needed to achieve saturation of themes (i.e., the point in the study when no new themes are identified in the data). As past research has identified treatment barriers for tics among young people with TS [10], participants who were in the process of receiving treatment or who have not received treatment at the time of the interview were included in the analysis. This allowed exploration of young peoples’ perceptions of access to care and perceived barriers towards the use of different treatments for tics.

### Data collection

#### Survey of parents

Target sample size was 300 participants based on Parker et al. [21]. The survey was designed to collect information about parents’ perceptions of treatment for their children’s tics. Survey questions were developed based on existing literature [8,13,10,17-19] and revised and piloted by experts working with this population and service users. The survey included forced choice tick box responses (e.g., “yes” or “no”), rating scales, ranking scales and text boxes for open comments. Automatic skip patterns were used to move participants past questions that were not applicable. Areas covered by the survey included: medication for tics; behavioural interventions for tics; and desired outcomes of treatment. On average, the survey took approximately 30 minutes to complete.

For the child’s clinical characteristics, parents were asked to report the child’s age of tic onset, diagnosis of tic disorders and diagnosis of coexisting conditions. Open-ended questions probed responses to access to treatment and diagnosis of TS. Parents and carers rated the impact of tics on their child’s self-esteem, social relationships and academic performance using the overall impairment scale of the Yale Global Tic Severity Scale (YGTSS) [22], adapted for online self-report.

Parents’ views on commonly used drugs (clonidine, risperidone, aripiprazole, sulpiride, haloperidol, olanzapine, quetiapine, pimozide, clonazepam and lorazepam) were sought, plus an option to comment on non-specified drug treatment. The 10 drugs were selected based on a recent survey of choice of medication for tics among members of the European Society for the Study of TS (ESSTS) [8] and our expert panel reports of current prescribing practices in the UK.

Participants were presented with a brief description of the comprehensive behavioural intervention for tics (CBIT) derived from the therapist guide for behavioural intervention for children and adults [23]. The description included components of habit reversal and function-based intervention (i.e., contingency management). If this treatment was not reported, parents were asked if they would like their child to be offered behavioural intervention for tics. Parents had the option to use text boxes for additional comments throughout the survey.

Finally parents were asked to rank, from most important to least important, seven potential positive outcomes of treatment for tics. In addition, parents were presented with two open-ended questions: i) “What would you most hope a treatment for tics would do for your child?”; and ii) “Are there any other benefits from treatment that you would like to see?”.

#### Interviews with young people

An interview schedule was developed in consultation with the expert panel and used to guide exploration of young people’s thoughts and experiences concerning services and treatments for tics. For young people who had not received medication and/or behavioural intervention for tics, the interview schedule included a short verbal description of each of these treatments.

Interviews were conducted by telephone or, where practical, face-to-face. Where the young person had been unable to recall specific information (e.g., name of a drug), permission was sought to check with the parent. Interviews, which lasted, on average, 40 minutes (range 20 to 73) were digitally recorded and transcribed verbatim following completion. Any personal identifiers were removed from the transcript.

### Data analysis

To integrate the qualitative data of the interviews of young people with TS with the quantitative and qualitative data of the survey of parents a process of triangulation was used, where the data from interviews and the data from the survey were analysed separately and the results drawn together where appropriate [24]. This permitted the examination of agreement, complementarity or disagreement [24] between the findings from the interviews with young people with TS and the survey of parents.

The study reported here adhered to the standards of reporting qualitative research (RATS) [25].

#### Parents’ survey

The survey data were analysed using SPSS v19 [26]. The numerical data reported were calculated based on the number of responses to each item and exclude missing values. Text data from open-ended questions were analysed using an inductive content analysis approach. Content analysis allows organisation of a large amount of text into fewer and meaningful categories [27]. The categories were developed by reading the participants’ responses to gain an overall sense of the data; developing codes to capture key concepts; labeling the codes, organising the codes based on commonalities; and finally defining the categories [27].

#### Interview analysis

The interview data were analysed using thematic analysis [28]. The purpose of the analysis was to explore young people’s needs and perceptions of treatments for tics. The themes were developed inductively because little is known about young people’s perceptions of different treatment strategies for tics. This inductive approach is used when past research in a topic is scarce and it is not possible to develop theory or research driven themes [28]. To become familiar with the data, the interview transcripts were read repeatedly and ideas about key aspects of the data were noted down to develop initial codes. These codes helped to organise the data into broader categories and they were revised and combined to identify possible themes. The themes and subthemes were discussed and refined following discussion with a qualitative expert leading the study (CG). In order to establish reliability a code book was developed which, for each theme, listed: i) the descriptive label; ii) the definition of the theme; iii) pointers to look for when identifying the theme; iv) exclusions to the theme; and v) a sample extract [28]. To establish the reliability of the coding one or two extracts from each of the themes and subthemes (total *n* = 26) were presented, unlabelled, together with the code book to a qualitative researcher (RW) who was not involved in the study. The researcher, who was blind to the original coding, used the codebook to code themes and subthemes. Her ratings were compared to the original coding by the study research fellow (JC) and agreement was excellent (92.3%, 24/26). Analysis of the interview data revealed eight superordinate themes relating to the young people’s experiences of treatment and care for TS of which seven had subthemes.

## Results

### Characteristics of parent sample

A total of 358 respondents consented to participate in the survey of whom 297 answered at least one question on treatment utilisation. Two entries were identified as repeat respondents and were removed from the data analysis, resulting in 295 participants with usable data. Of the 295 participants, 276 (93.6%) reported that their child had received a diagnosis of TS, eight (2.7%) reported a diagnosis of one or more tic disorders (3 vocal tic disorder, 2 transient tic disorder and only 3 reported one or more tic disorders) and 11 (3.7%) did not report a diagnosis of TS or tic disorder or had missing values on these items. All 11 reported age of onset for tics and were included in the analysis.

As shown in Table 1, the majority of participants were biological mothers (92.2%, n = 237) and the mean age of the sample was 44 years (SD = 6.3). Parents and carers described young people’s demographic and clinical characteristics. Young people (234 males, 61 females) ranged in age from 5 to 17 (M = 12.4, SD = 3.0). The mean age of tic onset was 5.9 years (SD = 2.8, range 1 to 17) and the mean age at diagnosis of TS was 9.1 (SD = 2.7, range 3–17). On average, parents reported that in the last seven days their child’s tic-related impairment was mild (M = 2.3, SD = 1.5, range 0 to 5). Sixty-six percent had one or more co-occurring conditions. Frequently reported co-occurring conditions were obsessive compulsive disorder (35.9%, n =106), ADHD (30.2%, n = 89), anxiety (24.7%, n = 73), autism spectrum disorder (20.3%, n = 60), depression (10.8%, n = 32) and learning disability (9.8%, n = 29).

**Table 1:** **Characteristics of survey participants (** ***n*** **= 295)**

**Characteristic**	
Age in years, Mean (SD), Range	44.0 (6.3), 27-68
Relationship to the child, *n* (%)	
Mother	237 (92.2)
Father	18 (7.0)
Other	2 (0.8)
Marital status, *n* (%)	
Married/cohabitating	201 (78.2)
Other	56 (21.8)
Highest level of education, *n* (%)	
Did not complete secondary school/compulsory education	7 (2.7)
Secondary school	58 (22.7)
Further education (e.g., A-Level)	85 (33.2)
Undergraduate or postgraduate	106 (41.4)

### Characteristics of young people’s sample

Eighty four potential participants provided contact details and 61 replied to the initial contact. Of these, four were excluded; three because service or treatment for tics was provided outside the UK and one was too young for inclusion. Of the remaining 57, five declined participation when contacted to arrange an interview, six could not identify a convenient time and four failed to keep their interview appointment. These 15 cases did not differ significantly in age and gender from the 42 young people who were interviewed (40 by telephone, 2 face-to-face). Of the 42 young people with TS who took part in the interviews, the majority were males (76.2%, n = 32) and the median age of the sample was 13 years (range 10 to 17) (Table 2). Twenty-three (54.8%) participants had taken medication for tics (median number of drugs taken = 1, range 1 to5). Eight (19.0%) young people had received some form of behavioural treatment for tics (median number of sessions received = 4, range 1 to 20), of whom seven had also received medication [see Additional file 1 for the characteristics of each interviewee].

**Table 2:** **Characteristics of interview participants (** ***n*** **= 42)**

**Characteristic**	
Age in years, Mean (SD), Range	13.4 (2.1), 10-17
Sex, *n* (%)	
Male	32 (76.2)
Female	10 (23.8)
Ethnicity, *n* (%)	
White British	36 (85.7)
Mixed/Multiple ethnic groups	6 (14.3)
Co-occurring conditions as reported by child’s parent (may be more than one condition), *n* (%)	20 (47.6)
Attention-deficit/hyperactivity disorder	9 (21.4)
Obsessive compulsive disorder	7 (16.7)
Autism spectrum disorder	6 (14.3)
Anxiety	4 (9.5)
Other	5 (11.9)
Treatment for tics, *n* (%)	
Medication	23 (54.8)
Behavioural intervention	8 (19.0)
No medication or behavioural intervention	18 (42.9)
Medication for tics, *n* (%)	
Clonidine	12 (28.6)
Risperidone	11 (26.2)
Aripiprazole	10 (23.8)
Haloperidol	3 (7.1)
Diazepam	2 (4.8)
Lorazepam	2 (4.8)
Sertraline	2 (4.8)
Atomoxetine	1 (2.4)
Clonazepam	1 (2.4)
Methylphenidate	1 (2.4)
Penicillin	1 (2.4)
Procyclidine	1 (2.4)

Analysis of qualitative data from interviews with young people is presented below and linked, where appropriate, to survey responses and common themes which emerged from analysis of text box data from the parent survey.

### Need for access to informed and expert care

Young people spoke about the importance of receiving care from health professionals with a good understanding of TS, and about the challenges accessing specialist care. This was quite a strong theme expressed by 22 young people. Views clustered in three subthemes.

#### Perceived lack of understanding of TS among health professionals

This subtheme captures how some young people felt that health professionals have insufficient knowledge to recognise TS and limited training in providing adequate treatment. Some young people described attending different health services to receive treatment for tics because many of the services they had visited had a poor understanding of their condition.

“…most of the places we have been to about my Tourettes like it seems like no one actually knows about it, like we know more than them… when we go there they usually ask us about it more than we ask them” [P 40]

#### Difficulties accessing or maintaining specialist care

This subtheme describes how some young people felt that there were difficulties in receiving specialist treatment such as delays, cancellations or insufficient funding. This subtheme also captured young people’s perceptions that they had received little or no information about TS and tics from their health professionals.

“…After I got diagnosed two years after that, that’s when I started to get information about it. …I would have liked it to have been just more of a frequent thing when I actually needed the help more. Some sort of like guidance on things I could do, instead of just finding out that I had to deal with it all on my own.” [P 32]

#### Importance of receiving informed specialist care

This subtheme reflects how young people particularly valued receiving care from health professionals who had a good understanding of TS and tics. In relation to this, young people spoke about feeling understood and more confident, and they considered that knowledgeable health professionals could provide useful information to better understand their condition.

These views were strongly echoed in the survey data with parents. Text boxes probed parents’ perceptions of access to care and the diagnosis of TS and the resulting content analysis showed that almost 32% (94/295) of parents spontaneously reported that it was difficult to get a referral to specialist treatment and to access appropriate care. Of these parents, 30 commented on the limited understanding of TS and tics among general practitioners (e.g., “We were told by our GP he would grow out of this habit and we were given antihistamines”) [see Additional file 2]. Parents’ comments also revealed that 16.3% (48/295) felt that receiving a diagnosis of TS was a long and difficult process. Moreover, 14.9% (44/295) of parents described receiving little information or support when the child received a diagnosis of TS.

### Concerns and limitations about taking medication for tics

This theme captured young people’s negative perceptions of medication for tics based on their experience of taking medication or on their understanding of what this treatment involves. This was a major theme endorsed by 29 young people with three subthemes emerging.

#### Perceived adverse effects of medication

This subtheme captures how young people who took medication for tics felt that it caused adverse effects. These effects were associated with different types of medication and typically included drowsiness, tiredness, self-reported depression, nightmares, weight gain and a sense of not being oneself. For some young people adverse effects were the main reason for stopping taking medication or for changing to a different drug, even if they perceived an improvement in tics.

#### Perceived limited or lack benefit of medication for tics

This subtheme reflects how some young people who took medication felt that it left the tics unchanged, had a positive effect on tics for a limited period of time or that it worsened their tics.

“I was on a medication called risperidone for a while, which was helpful to start off with, it certainly had a noticeable effect but once I had got the level, my risperidone level sort of steady after about a year or after about six months it stopped being so effective so I went off it…” [P 29]

#### Concerns about taking medication for tics

This subtheme describes how young people who had not taken medication for tics perceived potential difficulties about taking drug treatment; including potential adverse effects, difficulties to remember to take the drug, the need to take medication for a prolonged period of time and the unpleasant taste of medication. In general, young people queried whether taking medication would harm their health.

Parents’ responses showed they shared young people’s concerns regarding adverse effects of medication. The survey showed that 54.7% (152/278) of parents mentioned that their child had taken medication for tics. As shown in Table 3, for most of the drugs examined, more than 46% of parents felt that the drug had moderate or severe adverse effects. Parents’ free text comments also suggested that parents were concerned about a wide variety of adverse effects including, most frequently, sleepiness, tiredness or drowsiness and weight gain [see Additional file 3]. The level of tic-related impairment was significantly higher among the group of parents whose child had taken medication for tics (M = 2.6, SD = 1.6) compared with the group of parents whose child had not (M = 1.9, SD = 1.2), *t*(273) = 4.2, *p* < .001.

**Table 3:** **Medication and behavioural interventions for tics as reported by parents (n = 295)**

			**Helpfulness**				**Adverse effects**			
			**Score (−2 to 2)**		**Responded “somewhat helpful” or “very helpful”**		**Score (0 to 3)**		**Responded “moderate” or “severe”**	
**Treatment**	***n***	**(%)**	**Mean**	**(** ***SD*** **)**	***n***	**(%)**	**Mean**	**(** ***SD*** **)**	***n***	**(%)**
**Medication**										
Risperidone	77	(27.7)	0.1	(1.3)	30	(39.5)	1.9	(1.1)	48	(63.2)
Clonidine	73	(26.3)	0.1	(1.1)	30	(41.1)	1.5	(1.1)	34	(46.6)
Aripiprazole	55	(19.9)	1.0	(1.0 )	38	(69.1)	0.9	(1.1)	14	(25.5)
Haloperidol	24	(8.7)	−0.1	(1.2)	6	(25.0)	1.9	(1.3)	17	(70.8)
Sulpiride	13	(4.7)	−0.2	(1.0)	2	(15.4)	1.3	(1.4)	6	(46.2)
Pimozide	7	(2.5)	0.3	(1.3)	3	(42.9)	1.7	(1.4)	4	(57.1)
Clonazepam	7	(2.5)	−0.1	(1.5)	3	(42.9)	2.0	(1.4)	5	(71.4)
Lorazepam	5	(1.8)	0.2	(1.3)	3	(60.0)	1.6	(1.5)	3	(60.0)
**Behavioural interventions for tics**	74	(25.9)	0.4	(1.0)	34	(48.6)	0.4	(0.8)	8	(11.4)

Helpfulness scale: −2 = unhelpful – tics got a lot worse; −1 = unhelpful – tics got a bit worse; 0 = tics stayed the same; 1 = somewhat helpful; 2 = very helpful. Adverse effects scale: 0 = none; 1 = mild; 2 = moderate; 3 = severe.

### Positive experiences of medication for tics

This theme describes how young people who had taken medication for tics felt that it helped them to reduce their tics or to have better control over them. For some young people, medication for tics allowed them to feel less self-conscious about their tics and to disguise them better when in public. The responses of 18 young people endorsed this theme.

More than a third (n = 7) of young people who endorsed this theme commented specifically on aripiprazole.

“I find aripiprazole helps quite a lot actually I am not as bad as I was. I don’t have many tics during the day or anything anymore.” [P 10]

In the online survey, parents also identified that medication could be helpful for the child’s tics. However, parents’ ratings of perceived helpfulness of medication were generally moderate. An exception to this was aripiprazole (see Table 3). In the interviews, of the 10 young people who had taken aripiprazole, six also described adverse effects, the most frequent being drowsiness or sleepiness (two young people reported dizziness, one shakiness and one drooling) but only one child had stopped taking it and this was because it had little positive effects rather than because of side effects.

### Behavioural interventions for tics is a ‘natural’ intervention that could be incorporated into daily life

Positive perceptions of behavioural interventions for tics were captured by this theme, expressed in two subthemes.

#### Positive experiences of behavioural interventions for tics

This subtheme describes how young people who had received behavioural interventions for tics felt that it helped them to better recognise, control or manage their tics. Of the eight young people who had received behavioural interventions for tics, six interviews included this subtheme. Although the treatment process was often described as long, young people felt that it was generally helpful.

“…it took me a while but now it works more… I don’t have to think oh I must bite my lip I just do it without thinking, but at the time I was just like oh this isn’t going to work but it got better.” [P 5]

#### Perceived potential helpfulness of behavioural interventions for tics

This subtheme reflects how young people who had not received behavioural interventions for tics generally held positive views about this treatment. Of the 34 young people who had not received behavioural interventions for tics, 15 were represented in this subtheme. Young people felt that this treatment involved learning and practising behaviours that were similar to some of the behaviours they have used to control their tics, and they were described as “natural”.

### Limitations of behavioural interventions for the treatment for tics

This theme captures young people’s negative perceptions of behavioural interventions for tics based on their direct experience of this intervention or on their understanding of what this treatment involves. This theme consists of two subthemes.

#### Negative experiences of behavioural interventions for tics

This subtheme describes how young people who had received behavioural interventions for tics felt that it was unhelpful and difficult to engage with. Of the eight young people who received behavioural interventions for tics, the responses of six endorsed this theme. Some young people felt that it was difficult to execute competing responses for motor or vocal tics, and one young person felt that behavioural interventions was changing “this to that” and remembered developing a tic from a competing response.

#### Perceived potential difficulties of behavioural interventions for tics

This subtheme reflects how young people who had not received behavioural interventions for tics felt that this treatment would take long, considerable effort from their part or much support from others. Of the 34 young people who had not received behavioural interventions for tics, 14 perceived at least some limitations. Some young people felt that it would be difficult to remember to do the treatment exercises, that they could interfere with daily activities or be ineffective for “strong” tics.

In the online survey, 25.9% (74/286) of parents reported their child had received a behavioural intervention but in around half of these cases (36/69) the child received fewer than five sessions. Compared with parents whose child received fewer than five sessions and those who reported between five and 10, parents with more than 10 sessions had the highest scores on perceived helpfulness of behavioural interventions, yet these differences did not reach significance at *p* < .05. A higher proportion of parents whose child had received behavioural interventions for tics reported that their child had taken medication, χ^2^(1, n = 278) = 8.9, *p* < .01.

Eight percent (6/74) of parents commented on the helpfulness of the treatment and a similar number of parents identified limitations [see Additional file 4]. Parents (9.5%, 7/74) also commented on how the young person’s age and tic severity influenced treatment engagement and outcome . Of note, 6.8% (5/74) of parents described difficulties receiving behavioural interventions for tics from knowledgeable treatment providers (e.g., “More training needed for professionals… and I speak as both parent and professional!”).

In the interviews with young people, those who had not received behavioural interventions for tics had both negative and positive views on this treatment. In contrast, in the online survey most parents whose child had not received behavioural intervention for tics seemed to have positive perceptions of this treatment. Among parents whose child had not received behavioural interventions, the majority (76.3%, 148/194) wanted their child to be offered this treatment and felt that knowledge and availability of this treatment were limited (22.3%, 33/148) (e.g., “My daughter has asked time and again for this kind of help but it isn’t available in X”) [see Additional file 5]. Moreover, 10.8% (16/148) of parents expressed an interest in behavioural interventions, particularly as an alternative to medication.

Among parents who did not desire behavioural interventions for their children, 19.6% (9/46) felt that their child did not need this treatment because the tics were currently minimal or manageable [see Additional file 5]. Another 19.6% (9/46) felt that behavioural interventions would not suit their child and 8.7% (4/46) commented that these interventions were unavailable when the child needed treatment.

### Desire for a treatment to stop or reduce tics and the urge to tic

Two thirds of young people (n = 28) spoke about what they hoped a treatment could offer them. Although commonly young people wanted a treatment to reduce tics, reducing the urge to tic emerged as a second important subtheme.

#### Wanting to stop or reduce tics

This subtheme describes how young people would like their tics to be reduced, stopped, taken away or eliminated.

#### Wanting to reduce urges to tic

Reflects how for some young people the urge to tic was perceived as uncomfortable or painful, and reducing it was seen as an important outcome of treatment.

“I would like something that would kind of stop the urge to do it all the time…” [P 34]

Interestingly parents did not identify controlling the urge to tic as one of their desired outcomes of treatment.

### Need to manage emotional responses associated with tics

During the interviews young people also spoke about wanting a treatment that could help them to reduce or manage negative emotions associated with tics. This theme was endorsed by 19 young people and it consists of two subthemes.

#### Negative emotions as contributing to tics

This subtheme describes how young people spoke of feeling worried, anxious, or stressed in relation to their tics. Some young people remembered experiencing these emotions in different situations, particularly social situations, and considered that these emotions worsened their tics.

“…it makes me worried and then because I am worried I do it [tic] more and then because I am doing it more and people are looking I do it even more than that.” [P 19]

#### Interest and attempts to reduce negative emotions associated with tics

This subtheme captures young people’s efforts to reduce self-reported anxiety-related emotions that were associated with tics. For some young people, these emotions were described as more troublesome than tics and they spoke about trying to find ways to reduce them. Some young people described wanting a treatment that could help them to feel relaxed and calmed.

### The importance of gaining a sense of control over TS

Half of the young people (n = 21) spoke about experiencing little or no control over tics and identified a sense of control as an important outcome of treatment.

#### Perceived lack of control over TS and tics

This subtheme reflects how some young people felt that their tics could “take over”; as if these had their own will or personality.

#### Need to gain control over TS and tics

This subtheme describes how young people tried or would like to stop their tics at their instigation. They felt that this would allow them to engage in other activities, such as school activities, “without having to think my tics are going to get in the way” [P 23].

In the survey, parents ranked “reduction in severity and frequency of your child’s tics” as the most important desired outcome of treatment [see Additional file 6]. This was confirmed in approaching half the text comments [see Additional file 7]. Again, in line with the young people’s priorities, almost a quarter (64/295) of parents hoped that treatment for tics would help the child to reduce negative emotions associated with tics, which were often described as worries, anxiety and frustration (e.g., “…stop him feeling anxious… Let his body have some peace and be able to relax without ticking”), and 19.3% (57/295) felt that a treatment should help the child to control the tics [see Additional file 7].

## Discussion

This study explored perceptions of treatments for tics by conducting in-depth qualitative interviews with young people with TS and a mixed-methods survey of parents’ views on their child’s treatment for tics. There were many commonalities between the views of the separate groups of parents and young people. Young people and parents expressed strong concerns that health professionals have limited knowledge of TS and this was often associated with difficulties accessing and receiving evidence-based treatment. Young people and parents described the benefits of medication for tics, aripiprazole being favourably perceived, yet both groups also described concerns about adverse effects of medication and these were a common explanation for stopping medication among young people. Access to behavioural interventions for tics seemed to be limited and young people and parents had mixed views on these interventions, yet they were generally welcomed as having few adverse effects. Reducing or stopping tics was a desired outcome of treatment for young people and parents, but so was gaining a sense of control over tics and reducing or managing negative emotions which families attributed to being associated with tics. Young people particularly highlighted the need to manage the discomfort and distress associated with the urge to tic but this was not recognised by parents.

The findings of this study suggest that young people with TS and parents have had difficulties getting a diagnosis of TS, getting referred to specialist treatment and receiving appropriate treatment for tics. Consistent with past research that has found delays in the diagnosis of TS [17], the survey of parents identified an average delay of three years between age of tic onset and age of diagnosis of TS. Parents’ comments further suggested that getting a referral to specialist treatment was a long and difficult process. In many cases health professionals, particularly at primary care level, were perceived as having a poor understanding of tics and there were many examples of insensitive or ill-informed communications. In relation to this, in the interviews young people described visiting many services in attempts to receive treatment for tics and perceived that their condition was not well understood among health professionals. A previous study showed that approximately a quarter of health professionals lacked general knowledge of TS and only 52% were aware of its diagnostic criteria [29]. Moreover, a recent case note study of children with TS presenting at a UK non-specialist service found that at first consultation 76.5% of GPs thought that the symptoms did not indicate TS or tic disorders, and only 12.5% of GPs referred the child to a specialist service [30]. Current clinical guidelines recommend that health professionals work closely with patients, parents and other key carers in providing information about tics and their treatment [13,15,16]. However, in addition to difficulties with diagnosis and access difficulties, the findings of the present study indicate that some young people who had received medication or behavioural interventions for tics perceived that health professionals had insufficient training in providing treatment.

Consistent with previous survey studies, medication was a frequently reported treatment for tics among young people [9,10] and risperidone, clonidine and aripiprazole were the three most commonly reported drugs [11]. Some young people and parents had positive perceptions of medication. For parents, aripiprazole was generally perceived as helpful for the child’s tics and with few adverse effects. Children also perceived that aripiprazole was effective and it also appeared to be well tolerated although, since the doses of each target drug were not known, it is not possible to determine the impact of dosage. It may be that aripiprazole is better tolerated than other antipsychotic medication. There is some evidence suggesting that aripiprazole might be an effective treatment in reducing tics [31] and this has been confirmed in a recent randomised trial [32]. Young people had varied experiences of medication for tics and benefits of medication were generally described in terms of a reduction of tics, better control of tics and feeling less self-conscious about tics. Adverse effects, however, were commonly described by young people and parents. For young people adverse effects varied, were experienced with different drugs and were a common reason for stopping medication which highlights the importance of informed choice with regard to medication.

The study suggests that access to behavioural interventions is limited and that young people and their parents have mixed views about this treatment; however, a common theme for young people and parents was the lack of adverse effects. A previous US survey of parents of young people with TS suggested only 23.6% received behavioural treatment [10]: very close to the 25.9% reported by parents in the present study. In addition to limited availability the results of the parent survey and the interviews with young people suggest that many may not receive sufficient “dose” of behavioural intervention, with parents reporting that most of those who receive this treatment get fewer than five sessions and young people reported a median of four sessions. Eight or more sessions have been recommended for CBIT [23] and 12 for exposure and response prevention (ER) which is an extension of habit reversal [33]. It is unclear why relatively few participants in the present study had received behavioural interventions for tics but it has been suggested [34] that this could be due to health professionals’ lack of knowledge regarding implementation [29] and difficulties finding treatment providers [10]. Parents’ comments indicated that 22% of those who wanted behavioural interventions for their child’s tics felt that health professionals lacked awareness or training in this approach.

Some parents and young people felt that behavioural interventions were helpful for managing tics, whereas others reported difficulties engaging with this intervention and a few parents and young people described negative reactions such as attributing a new tic to treatment exercises. Young people’s negative perceptions of behavioural interventions seemed to be linked to the duration, motivation and effort needed to practise this treatment, rather than to adverse effects. Using data from two large CBIT trials of children and adults, a recent study examined frequently reported concerns of CBIT including the claim that this intervention requires considerable effort for patients and that it contributes to tic substitution [14]. The study suggests that CBIT may not require considerable effort for patients since attrition in the trials was low (9.5% to 13.9%) and comparable to placebo-controlled medication trials [14]. In addition, the study suggests that CBIT does not result in tic substitution since in both trials there was an improvement in the YGTSS scores [14]. Since the present study examined perceptions of treatment for tics, its findings suggest that these concerns exist in some young people with TS and their parents, and that health professionals may need to address these concerns when providing behavioural interventions. There is evidence that behavioural interventions for tics can be effective in reducing tics [35] and current clinical guidelines suggest such interventions as a first line treatment [12-14]. To increase the accessibility of behavioural interventions for tics other forms of delivery could be further investigated, such as videoconference for which there is some preliminary evidence of its effectiveness [36].

A novel feature of these results was the importance that both parents and young people placed on managing the negative emotions associated with TS which were generally described as worries, anxiety and stress. Past research suggests that anxiety, stress and tension are linked to tic exacerbation [37]. The present study seems consistent with this, but further suggests that young people and parents hope that a treatment for tics helps them to manage anxiety-related symptoms. Treatment outcome measures could incorporate the evaluation of anxiety and stress related to tics. Gaining a sense of control over tics was also mentioned by young people and parents. In relation to this, a previous qualitative study with young people with TS identified that young people felt that they needed to control their tics [4]. For young people, help to deal with the urge to tic was important but parents did not recognise this outcome as important. In this regard, ER is one behavioural intervention for tics that has shown to reduce the urge to tic by teaching the child to resist the urge as long as possible [38]. Research is needed to explore the scope of tailored behavioural interventions to deliver treatment outcomes that are important to young people.

### Strengths and limitations

The survey had a large sample size (*n* = 295) which closely approximated the minimum of 300 recommended by Parker et al. [21] to ensure valid results. By using an anonymous online survey, parents had the opportunity to describe both positive and negative perceptions of treatment for tics, whereas the individual interviews with young people might have reduced the potential influence of other young people’s opinions if data were collected in groups. Although only 20% of the children who were the subject of the survey and 23% of young people who were interviewed were female this is a close reflection of the 20%-25% female prevalence observed in representative samples of children with TS [39]. Participants were recruited through Tourettes Action (TA); membership or communication with TA may reflect a proactive population with common views on specific treatments for tics and the findings, based on a UK population, may not generalise to all young people with TS. Although we would have liked to recruit more participants who had direct experience with behavioural interventions for tics, the relatively small proportion of parents and young people who received this treatment might reflect its limited availability in a UK population. Recall bias may have influenced parents’ and young people’s perceptions of treatments for tics, particularly if the young person received treatment several years before data collection. Some young people chose to be interviewed with their parent present which might have influenced responses. However, young people seemed to talk openly and to consult the parent only when they were unable to remember details of treatment, such as a drug name or the time when they received an intervention. Interviews were conducted and analysed by one researcher (JC) who had previous experience of working with children and young people but who was not an expert on TS. As someone who not involved in the setting of the original research questions or in clinical care, arguably interactions with participants were not strongly driven by the interviewer’s preconceptions about treatment.

## Conclusions

Many young people and their parents in the present study perceived that TS and tic treatments are generally not well understood among health professionals. Because they described difficulties accessing specialist treatment and appropriate care, it is in the interest of young people and their parents that professional training expands knowledge and awareness of TS and tic treatments among health professionals, particularly at primary care level. Since medication was a common treatment for tics and many parents and young people perceived adverse effects of medication, young people and their families may value receiving clear information regarding the rationale for using medication and its potential adverse effects. Behavioural interventions for tics were generally perceived as having little or no adverse effects, but access to and knowledge of this treatment seemed limited and further research could investigate if its accessibility could be increased through the use of technology. Stopping or reducing tics was clearly a desired outcome of treatment, but so was managing anxiety related to tics and gaining a sense of control over tics, which could be incorporated in the evaluation of treatment outcomes.

## Additional files

Additional file 1:
**Interviewee identifying codes and characteristics.** For each of the 42 young people who participated in the qualitative interviews, this table provides demographic and clinical information and the identification code that was assigned. Additional file 2:
**Access to care and diagnostic process as described by parents (N = 295).** Based on parents’ text responses to survey questions about access to care and the diagnostic process, this table displays the categories derived from the content analysis, the distribution of responses across these categories and example responses. Additional file 3:
**Adverse effects of medication as described by parents (N = 295).** Based on parents’ text responses to survey questions about medication for tics, this table displays frequently described adverse effects and example responses. Additional file 4:
**Perceptions of behavioural interventions for tics among parents whose child has received this intervention (n = 74).** Based on parents’ text responses to survey questions about behavioural interventions for tics (among parents whose child has received this intervention), this table displays the categories derived from the content analysis, the distribution of responses across these categories and example responses. Additional file 5:
**Perceptions of behavioural interventions for tics among parents whose child has not received this intervention (n = 203).** Based on parents’ text responses to survey questions about behavioural interventions for tics (among parents whose child has *not* received this intervention), this table displays the categories derived from the content analysis, the distribution of responses across these categories and example responses. Additional file 6:
**Parental ranking of desired outcomes of treatment.** Based on parents’ responses to a survey ranking question about desired outcomes of treatment for tics, this table displays parents’ selected outcomes of treatment ranked from most important to least important. Additional file 7:
**Desired outcomes of treatment for tics as described by parents (N = 295).** Based on parents’ text responses to questions about desired outcomes of treatment for tics, this table displays the categories derived from the content analysis, the distribution of responses across these categories and example responses.
