# Supplementary material for: Perceptions of treatment for tics among young people with Tourette syndrome and their parents: a mixed methods study
Source: BMC Psychiatry. 2015 Mar 11;15:46. doi: 10.1186/s12888-015-0430-0 (PMC4359496; doi:10.1186/s12888-015-0430-0)
Supplement: Additional file 1: — Interviewee identifying codes and characteristics. For each of the 42 young people who participated in the qualitative interviews, this table provides demographic and clinical information and the identification code that was assigned. [file 12888_2015_430_MOESM1_ESM.docx]

# Additional files

### Additional file 1 – Interviewee identifying codes and characteristics

|  |  |  |  |  |  | **Co-occurring conditions** | | | | |
| --- | --- | --- | --- | --- | --- | --- | --- | --- | --- | --- |
| **Participant identification code** | **Gender** | **Age in years** | **Has taken medication for tics** | **Has received some form of behavioural intervention for tics** | **Other treatment/intervention for tics** | **ADHD** | **OCD** | **ASD** | **Anxiety** | **Other** |
| 1 | Male | 14 | Yes | No | Cognitive Behavioural Therapy; relaxation training | No | Yes | No | No | No |
| 2 | Male | 11 | Yes | Yes |  | No | No | No | No | No |
| 3 | Male | 12 | No | No |  | No | Yes | No | Yes | No |
| 4 | Male | 17 | No | No |  | Yes | No | No | No | No |
| 5 | Male | 15 | Yes | Yes |  | No | No | No | No | No |
| 6 | Female | 14 | Yes | Yes |  | No | No | No | No | No |
| 7 | Female | 11 | Yes | No |  | No | Yes | No | No | No |
| 8 | Male | 17 | Yes | No |  | No | Yes | Yes | No | Yes |
| 9 | Female | 17 | Yes | No |  | No | No | No | No | No |
| 10 | Female | 17 | Yes | No | Massage | No | No | Yes | No | No |
| 11 | Male | 15 | Yes | No | Relaxation training | Yes | No | No | Yes | No |
| 12 | Male | 17 | Yes | No |  | Yes | No | No | Yes | No |
| 13 | Male | 13 | No | No | Psychotherapy | No | No | No | No | No |
| 14 | Male | 11 | Yes | No |  | No | No | No | No | No |
| 15 | Female | 13 | Yes | No |  | No | No | No | No | Yes |
| 16 | Male | 13 | No | No |  | No | No | No | No | No |
| 17 | Male | 10 | No | No | Auditory listening programme | No | No | Yes | No | Yes |
| 18 | Male | 16 | Yes | Yes |  | No | No | No | No | No |
| 19 | Male | 12 | Yes | No | Relaxation training | No | No | No | No | No |
| 20 | Male | 12 | No | No |  | No | No | No | No | No |
| 21 | Male | 11 | No | No |  | Yes | No | No | No | No |
| 22 | Male | 16 | Yes | No |  | No | Yes | No | Yes | No |
| 23 | Male | 13 | No | No |  | No | Yes | No | No | No |
| 24 | Female | 13 | Yes | No |  | Yes | No | No | No | Yes |
| 25 | Male | 13 | No | No |  | No | No | No | No | No |
| 26 | Male | 11 | No | No |  | No | No | No | No | No |
| 27 | Male | 15 | Yes | No | Anger management | No | No | No | No | No |
| 28 | Male | 13 | No | No |  | No | No | No | No | No |
| 29 | Male | 16 | Yes | No | Relaxation training | No | No | No | No | No |
| 30 | Male | 12 | Yes | No |  | No | No | No | No | No |
| 31 | Male | 12 | Yes | Yes |  | No | No | No | No | No |
| 32 | Male | 15 | No | No |  | No | No | No | No | No |
| 33 | Male | 10 | No | No |  | No | Yes | No | No | No |
| 34 | Female | 11 | No | No |  | No | No | No | No | No |
| 35 | Female | 12 | No | No | Play therapy; Cognitive Behavioural Therapy; counseling | No | No | No | No | No |
| 36 | Female | 13 | No | No |  | No | No | No | No | No |
| 37 | Male | 13 | No | No | Training to suppress tics (not habit reversal) | Yes | No | No | No | No |
| 38 | Male | 17 | Yes | Yes |  | Yes | No | No | No | No |
| 39 | Male | 14 | Yes | Yes |  | No | No | No | No | No |
| 40 | Male | 11 | No | No |  | Yes | No | Yes | No | No |
| 41 | Female | 13 | Yes | No |  | Yes | No | Yes | No | Yes |
| 42 | Male | 12 | No | Yes | Relaxation training | No | No | Yes | No | No |
| ADHD = attention-deficit/hyperactivity disorder. OCD = obsessive compulsive disorder. ASD = autism spectrum disorder. | | | | | | | | | | |
