# Supplementary material for: Perceptions of treatment for tics among young people with Tourette syndrome and their parents: a mixed methods study
Source: BMC Psychiatry. 2015 Mar 11;15:46. doi: 10.1186/s12888-015-0430-0 (PMC4359496; doi:10.1186/s12888-015-0430-0)
Supplement: Additional file 2: — Access to care and diagnostic process as described by parents (N = 295). Based on parents’ text responses to survey questions about access to care and the diagnostic process, this table displays the categories derived from the content analysis, the distribution of responses across these categories and example responses. [file 12888_2015_430_MOESM2_ESM.docx]

# Additional files

### Additional file 2 – Access to care and diagnostic process as described by parents (N = 295)

| **Category** | **Number of parents in each category** | **Percentage (overall sample)** | **Example comment** |
| --- | --- | --- | --- |
| Difficulties with getting referred to specialist treatment and accessing appropriate care | 94 | 31.9% | “We went to a GP at our local practice three times within the first year and were told it was a phase and nothing to worry about - despite the fact that the tics (vocal and motor) were frequent and having a pronounced impact on my son's school and home life.” |
| Delay and difficulty with getting diagnosis | 48 | 16.3% | “Frustrating for us as parents that it took so long. Once diagnosed everything made sense and we felt that we could help our son.” |
| Lack of support or poor communication with regard to diagnosis | 44 | 14.9% | “They never at any point sat us down and explained a diagnosis, they just dropped it into a casual conversation one day as if I knew. If you have cancer or something a doctor sits with you and explains everything, with mental health it seems different. WHY?” |
| Straightforward for diagnosis, particularly if in the system | 39 | 13.2% | “Once CAMHS [Child and Adolescent Mental Health Services] were involved I felt my concerns were listened to and respected and further monitoring and assessments took place leading to the diagnosis” |
| Straightforward to access treatment or specialist care | 28 | 9.5% | “Family doctor was very supportive and immediately referred us to a local CAMHS.” |
| Usefulness of diagnosis | 8 | 2.7% | “It was a relief to finally be able to understand the syndrome and come up with a plan to help it” |
| Difficulty for parents recognising Tourette syndrome | 8 | 2.7% | “I originally thought it was just a habit he was going through but his tics continued to get worse.” |
| Other | 4 | 1.4% |  |
