# Supplementary material for: Perceptions of treatment for tics among young people with Tourette syndrome and their parents: a mixed methods study
Source: BMC Psychiatry. 2015 Mar 11;15:46. doi: 10.1186/s12888-015-0430-0 (PMC4359496; doi:10.1186/s12888-015-0430-0)
Supplement: Additional file 3: — Adverse effects of medication as described by parents (N = 295). Based on parents’ text responses to survey questions about medication for tics, this table displays frequently described adverse effects and example responses. [file 12888_2015_430_MOESM3_ESM.docx]

# Additional files

### Additional file 3 – Adverse effects of medication as described by parents (N = 295)

| **Medication** | **Received medication** | **Parents commenting on adverse effects** | | **Frequently described adverse effects** ^a^  (*n* of parents describing adverse effect; % among those who received medication) | **Example comment** |
| --- | --- | --- | --- | --- | --- |
|  |  | ***n*** | **(%)** |  |  |
| Risperidone | 77 | 32 | (41.6) | Weight gain/increase of appetite (*n* = 9; 11.7%) | “He only took it for 3 weeks as he developed a monstrous appetite and gained rapid weight…” |
|  |  |  |  | Other (*n* = 21; 27.3%) | “My son became more agitated and impulsive on this medicine. It was a nightmare!” |
| Clonidine | 73 | 17 | (23.3) | Depression (*n* = 5; 6.8%) | “Our son became depressed on clonidine - extremely weepy…” |
|  |  |  |  | Sleepy/tired/drowsy (*n* = 5; 6.8 %) | “He was very sleepy on this medicine and it didn't help.” |
|  |  |  |  | Other (*n* = 10; 13.7% ) | “Make his blood pressure very low and was told by hospital doctor to stop taking it” |
| Aripiprazole | 55 | 14 | (25.5) | Sleepy/tired/drowsy (*n* = 4; 7.3%) | “He has been on this med for approx. 16 months. Was very sleepy to begin with.” |
|  |  |  |  | Weight gain/increase of appetite (*n* = 4; 7.3%) | “Have to watch what he eats, makes him hungry…” |
|  |  |  |  | Other (e.g. nausea, akathisia/ tremor) (*n* = 8; 14.5%) | “…she had very bad nausea and unable to eat in the beginning but that has now gone sometimes she gets a bit shaky when taking it mainly shaky hands…” |
| Haloperidol | 24 | 12 | (50.0) | Sleepy/tired/drowsy (*n* = 5; 20.8%) | “…is very sleepy at times, he often falls asleep at school.” |
|  |  |  |  | Other e.g. dystonia (*n* = 6; 25.0%) | “Only took this medication for a couple of days. Caused breathing problems and muscle spasms.” |
| Sulpiride | 13 | 5 | (38.5) | Sleepy/tired/drowsy (*n* = 2; 15.4%) | “Became easily fatigued when he first started taking medication…” |
|  |  |  |  | Weight gain/increase of appetite (*n* = 2; 15.4%) | “…she put on weight…” |
|  |  |  |  | Other (*n* = 3; 23.1%) | “Took it at age 6. Only took it for 10 days. Vomited every day he took it…” |
| Pimozide | 7 | 1 | (14.3) | Mixed (weight gain, insomnia) | “…caused weight gain and insomnia and tiredness in the day unable to get up in the morning also caused dizziness.” |
| Clonazepam | 7 | 2 | (28.6) | Mixed | “Only took on one occasion but had such a horrible almost hallucinogenic effect we didn’t use again” |
| Lorazepam | 5 | 3 | (60.0) | Sleepy/tired/drowsy (*n* = 2; 40.0%) | “Too sleepy unable to think” |
|  |  |  |  | Other (*n* = 2; 40.0%) | “Caused increase in anxiety…” |
