# Supplementary material for: Perceptions of treatment for tics among young people with Tourette syndrome and their parents: a mixed methods study
Source: BMC Psychiatry. 2015 Mar 11;15:46. doi: 10.1186/s12888-015-0430-0 (PMC4359496; doi:10.1186/s12888-015-0430-0)
Supplement: Additional file 4: — Perceptions of behavioural interventions for tics among parents whose child has received this intervention (n = 74). Based on parents’ text responses to survey questions about behavioural interventions for tics (among parents whose child has received this intervention), this table displays the categories derived from the content analysis, the distribution of responses across these categories and example responses. [file 12888_2015_430_MOESM4_ESM.docx]

# Additional files

### Additional file 4 – Perceptions of behavioural interventions for tics among parents whose child has received this intervention (n = 74)

| **Category** | **Number of parents in each category** | **Percentage** | **Example comment** |
| --- | --- | --- | --- |
| Child’s age and tic severity affect treatment engagement and outcome | 7 | 9.5% | “…my son wasn't very responsive - perhaps a little too young to receive the full benefit.”  “Difficult to encourage engagement when tics are mild but need to do so as when tics are more severe it's hard to learn the process.” |
| Perceived helpfulness of treatment | 6 | 8.1% | “The success varies, but compared with how my son behaved when he was first diagnosed, there is a definite improvement in his ability to manage the less severe episodes.” |
| Limitations of treatment | 6 | 8.1% | “My son found it very difficult to do the opposite response so at the time this treatment didn’t help him. Maybe in the future it may be an option again.” |
| Difficulties receiving informed and comprehensive treatment | 5 | 6.8% | “The psychologist was caring but inexperienced… The 2-hour journey to X by train was a little distressing for him and made his tics worse.” |
| Child is in early stages of treatment | 4 | 5.4% | “My son has recently started this treatment so it is too soon to know if it will help in the future.” |
| Other | 7 | 9.5% |  |
