# Supplementary material for: Perceptions of treatment for tics among young people with Tourette syndrome and their parents: a mixed methods study
Source: BMC Psychiatry. 2015 Mar 11;15:46. doi: 10.1186/s12888-015-0430-0 (PMC4359496; doi:10.1186/s12888-015-0430-0)
Supplement: Additional file 5: — Perceptions of behavioural interventions for tics among parents whose child has not received this intervention (n = 203). Based on parents’ text responses to survey questions about behavioural interventions for tics (among parents whose child has not received this intervention), this table displays the categories derived from the content analysis, the distribution of responses across these categories and example responses. [file 12888_2015_430_MOESM5_ESM.docx]

# Additional files

### Additional file 5 – Perceptions of behavioural interventions for tics among parents whose child has not received this intervention (*n* = 203)

| **Category** | **Number of parents in each category** | **Percentage** | **Example comment** |
| --- | --- | --- | --- |
| **Parents who would like to be offered behavioural interventions for tics (*n* = 148)** | | | |
| Limited knowledge and availability of treatment | 33 | 22.3% | “I have asked my GP for information about HRT [habit reversal therapy], which the paediatrician consultant who diagnosed my son had suggested. Although her advice to me was to Google it and do it myself. Currently waiting for GP to get back to me regarding this.” |
| Interest in treatment, particularly as an alternative to medication | 16 | 10.8% | “I would of liked to have been offered this as a treatment option, my son has mild to moderate tic, we declined the use of medication because the side effects would outweigh the benefit…” |
| Treatment would be appropriate when child is older | 6 | 4.1% | “Now he is older I think it would be helpful. When he was younger (eg 11) he just said he would tic whatever. Now he has more interest in minimising his tics in public. He is now more aware that he has some control. The treatment offered should change with age.” |
| In the process of receiving treatment | 5 | 3.4% | “We have just been offered this treatment for our son and are on the verge of being taught the HRT [habit reversal therapy] course.” |
| Other | 2 | 1.4% |  |
| **Parents who would not like to be offered behavioural interventions for tics (*n* = 46)** | | | |
| No need for treatment as tics are currently minimal or manageable | 9 | 19.6% | “Currently, my son manages his condition well and we are not seeking further help.”  “Son's tics are so mild he is more than happy to just live with them. He states they do not bother him, they make him who he is.” |
| Treatment would not suit the child | 9 | 19.6% | “She tics approximately every 4 seconds, these exercises would not benefit her.”  “The alternatives consume more mental and physical energy than the tics themselves - no solution for my son's style or level of tics.” |
| Treatment was unavailable when needed | 4 | 8.7% | “I did ask that my son be given these treatments, received a letter back from our local hospital to state that they didn’t have the resources and that when he had been diagnosed, 4yrs previous, it was noted that he didn’t suffer at school/socially due to his condition! That was before he was really aware of being any 'different' and the years of teenage worries! I have no faith at all in health services...” |
| Other | 3 | 6.5% |  |
