# Supplementary material for: Perceptions of treatment for tics among young people with Tourette syndrome and their parents: a mixed methods study
Source: BMC Psychiatry. 2015 Mar 11;15:46. doi: 10.1186/s12888-015-0430-0 (PMC4359496; doi:10.1186/s12888-015-0430-0)
Supplement: Additional file 6: — Parental ranking of desired outcomes of treatment. Based on parents’ responses to a survey ranking question about desired outcomes of treatment for tics, this table displays parents’ selected outcomes of treatment ranked from most important to least important. [file 12888_2015_430_MOESM6_ESM.docx]

# Additional files

### Additional file 6 – Parental ranking of desired outcomes of treatment

| **Desired outcome** | **Mean** | **(*SD*)** | **Mode** |
| --- | --- | --- | --- |
| Reduction in severity and frequency of your child’s tics | 1.8 | (1.3, n = 253) | 1 |
| Your child is better able to cope with his or her tics | 1.9 | (1.0, n = 252) | 2 |
| School is better able to manage your child's tics | 4.3 | (1.4, n = 253) | 3 |
| Your child gets on better with friends | 4.4 | (1.4, n = 253) | 5 |
| Your child gets on better at school | 4.7 | (1.4, n = 253) | 6 |
| Your family is better able to cope with your child's tics | 5.0 | (1.6, n = 253) | 7 |
| Your child can take part in more leisure activities | 6.0 | (1.3, n = 252) | 7 |
