# Supplementary material for: Perceptions of treatment for tics among young people with Tourette syndrome and their parents: a mixed methods study
Source: BMC Psychiatry. 2015 Mar 11;15:46. doi: 10.1186/s12888-015-0430-0 (PMC4359496; doi:10.1186/s12888-015-0430-0)
Supplement: Additional file 7: — Desired outcomes of treatment for tics as described by parents (N = 295). Based on parents’ text responses to questions about desired outcomes of treatment for tics, this table displays the categories derived from the content analysis, the distribution of responses across these categories and example responses. [file 12888_2015_430_MOESM7_ESM.docx]

# Additional files

### Additional file 7 – Desired outcomes of treatment for tics as described by parents (N = 295)

| **Category** | **Number of parents in each category** | **Percentage (overall sample)** | **Example comment** |
| --- | --- | --- | --- |
| Reduce or stop tics | 121 | 41.0% | “Bring them to a tolerable level or even stop them”  “Ideally I would like a treatment that would eliminate his tics with no side effects” |
| Manage or reduce negative emotions associated with tics | 64 | 21.7% | “Reduce feelings of anxiety and frustration”  “A feeling of being less anxious, so as to not worry about everything…” |
| Child controls/manages tics | 57 | 19.3% | “For him to be able to control them at his instigation”  “Give him control over them and be able to substitute one for a less noticeable/harmful type” |
| Increase self-esteem and confidence | 46 | 15.6% | “Help him overcome his lack of self-confidence.”  “Boost his self-esteem, make him feel 'normal' and not as inadequate in social situations” |
| Increase knowledge and acceptance of condition | 35 | 11.9% | “Just give us a better understanding of why they happen and how to deal with them”  “A place where doctors send children with tics where the practitioner understands what you are describing and can give you a straight answer would be nice” |
| Enhance general quality of life | 18 | 6.1% | “Raising of quality of life through an ability to spend time on other activities when time lots of time is spent ticking…” |
| Social integration | 16 | 5.4% | “…being able to have a good social life without being self-conscious about his condition” |
| Reduce physical pain associated with tics | 16 | 5.4% | “Reduce the pain caused by constant tics and jerks” |
| Other | 43 | 14.6% |  |
